# Gastric greater curvature plication combined with Nissen fundoplication compared to sleeve gastrectomy in patients with obesity and animal model

**DOI:** 10.1186/s12893-025-02960-3

**Published:** 2025-05-29

**Authors:** Chong Cao, Bo Xu, Yu Wang, Yikai Shao, Qiwei Shen, Xiaojian Fu, Rong Hua, Qiyuan Yao

**Affiliations:** 1 Center for Obesity and Hernia Surgery, Department of General Surgery, Huashan Hospital, Fudan University, Shanghai, 200040 China; 2 Department of Nursing, Huashan Hospital, Fudan University, Shanghai, 200040 China

**Keywords:** Gastric greater curvature plication, Nissen fundoplication, Sleeve gastrectomy, Bariatric surgery, Obesity

## Abstract

**Background:**

Gastric greater curvature plication combined with Nissen fundoplication (GGCP + Nissen) has been previously performed, but its efficacy remains uncertain.

**Methods:**

A single-center retrospective review was conducted on patients with obesity who underwent GGCP + Nissen or sleeve gastrectomy (SG) between January 2016 and December 2022. Both groups were matched for age, gender, and baseline BMI. In the animal experiments, GGCP + Nissen, SG, and sham procedures were performed on Goto-Kakizaki rats, a model of normal-weight rats with T2DM.

**Results:**

A total of 75 patients were included in this study, of whom 35 underwent GGCP + Nissen, and 40 underwent SG. Both groups were matched for age, gender, and baseline BMI. The percent total weight loss (%TWL) in the GGCP + Nissen and SG groups were 16.38 ± 3.69 and 25.05 ± 7.24 at 6 months (*P* < 0.05), 16.40 ± 4.96 and 26.85 ± 9.13 at 12 months (*P* < 0.05), and 12.46 ± 5.90 and 24.57 ± 8.61 at 24 months (*P* < 0.05), respectively. However, all 8 patients with preoperative reflux in the GGCP + Nissen group achieved complete resolution of symptoms postoperatively, whereas in the SG group, 10 patients developed new-onset reflux at 1 month, with 4 continuing to experience persistent symptoms at 24-month follow-up. In the animal experiments, both GGCP + Nissen and SG induced significant weight loss and improved glucose tolerance, with rats showing increased insulin sensitivity and secretion. However, the SG group performed better than the GGCP + Nissen group in terms of both weight loss and improvement of glucose tolerance.

**Conclusions:**

GGCP + Nissen was inferior to SG both in weight loss and improvement of glucose tolerance, although GGCP + Nissen could lead a substantial weight loss and improve GERD efficiently.

## Background

Bariatric surgery has been shown to be effective at mitigating or resolving many obesity-related health conditions. Sleeve gastrectomy (SG) and Roux-en-Y gastric bypass (RYGB) are the two bariatric surgeries most commonly performed to treat obesity. Both procedures can result in significant weight loss and improvements in metabolic health; however, they are associated with surgical risks and complications. Common complications include bleeding, infections, and emboli and thrombosis, while long-term complications include nutritional deficiency, gastrointestinal problems, and the potential need for additional surgery [1, 2].

Gastric greater curvature plication (GGCP) had been developed with the intention to reduce the complications caused by bariatric surgery. GGCP is a bariatric surgery that involves folding and suturing the greater curvature of the stomach to reduce its size and capacity. This results in a feeling of fullness after smaller meals which translates to a decreased in food intake and subsequent weight loss. The procedure is performed laparoscopically and does not require removal of stomach tissue or rerouting of the gastrointestinal tract. This makes it a less invasive and potentially safer alternative to more standard bariatric surgeries such as RYGB or SG [3, 4]. Additionally, due to the operation not requiring expensive surgical staplers, it lowers the surgical costs, making it more acceptable for patients.

GGCP with Nissen fundoplication (GGCP + Nissen) is a modification of the traditional GGCP procedure that includes Nissen fundoplication to prevent gastroesophageal reflux disease (GERD) and improve long-term weight loss outcomes [5, 6]. Obesity is a major risk factor in the development of GERD, the risk of which increases with body mass index (BMI) [7]. Although bariatric surgery is effective at improving obesity, some procedures, including SG and GGCP, may not improve reflux, and can lead to de novo GERD due to the destruction of the anti-reflux structure of the original gastric fundus [8, 9]. In the GGCP + Nissen procedure, Nissen fundoplication is performed in addition to standard GGCP, with the gastric fundus wrapped around the esophagus and sutured in place to create a valve that prevents the backflow of stomach contents into the esophagus. This procedure is generally considered safe and effective at relieving the symptoms of GERD, and most patients experience significant improvements in their quality of life [10, 11]. However, few studies have demonstrated the efficacy of GGCP in combination with Nissen. In this study, we enrolled patients with obesity who had a mean body mass index of approximately 30 kg/m^2^ who underwent GGCP + Nissen or SG to compare the clinical outcomes. Further, we used a rat model to evaluate the effects of GGCP + Nissen and SG on glucose metabolism. The overall aim of this study was to compare the outcomes of GGCP + Nissen with the outcomes of SG for the treatment of obesity and diabetes, all while keeping in mind the reflux issues suffered by the obese patient and how the procedures relate to the reflux.

## Methods

### Patients, surgical technique, and clinical assessment

This prospective study was conducted at the Center for Obesity and Metabolic Surgery, Department of General Surgery, Huashan Hospital, Fudan University, Shanghai, China, between January 2016 and December 2022. This study was approved by the Ethics Committee of our institution. Our study enrolled 75 patients. There were 35 patients suffering from obesity who underwent the GGCP + Nissen procedure; there were 40 patients suffering from obesity who underwent SG. The GGCP + Nissen cohort comprised 35 consecutively enrolled patients. A matched SG group (*n* = 40) was derived using age, gender and baseline BMI as covariates to minimize selection bias.

**Fig. 1:**
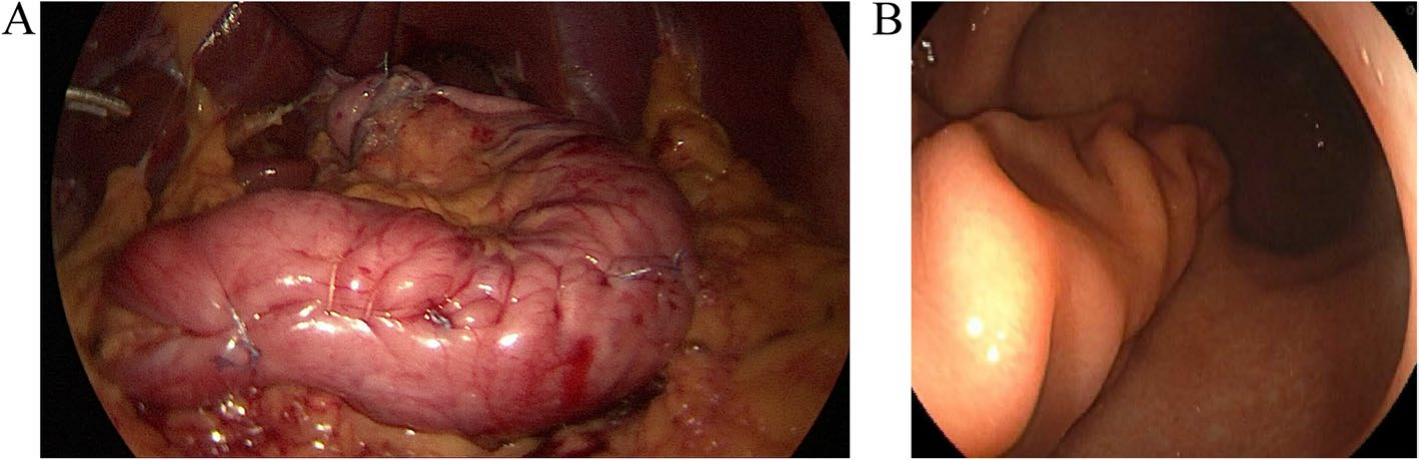
Gastric greater curvature plication combined with Nissen fundoplication in patient. **A** Laparoscopic vision. **B** Endoscopic vision

All surgeries were performed laparoscopically. The GGCP + Nissen and SG procedure were performed as previously described [5, 12]. For GGCP + Nissen, the greater curvature was mobilized with an ultrasonic dissector from 3 cm above the pylorus to the left diaphragmatic crus. The hepatogastric ligament was divided to expose the right crus, which was sutured with 2–0 non-absorbable sutures to snugly encircle the esophagus. A short-floppy Nissen fundoplication was created. From the Nissen’s inferior margin, the greater curvature was imbricated 3 cm from the lesser curvature using a continuous 3–0 barbed suture. Reinforcement was added with a continuous 2–0 non-absorbable suture extending from 3 cm above the pylorus to the fundus. For SG, the gastrocolic ligament was divided at the mid-greater curvature, and dissection proceeded cephalad to mobilize the fundus, exposing the esophagus and left diaphragmatic crus, then caudally to 2 cm above the pylorus. A 36 Fr bougie guided sequential stapling from 3 cm above the pylorus to 1 cm below the cardia. Hemostasis was achieved, and the staple line was reinforced with a 3–0 barbed suture. The picture of GGCP + Nissen was showed in Fig. 1.

Weight and height were measured before surgery and at 1, 6, 12, and 24 months postoperatively. Weight outcomes were presented as percentages of total body weight loss (%TWL). All patients completed the GERD-questionnaire (GERD-Q) score before surgery and at 1, 6, 12, and 24 months after operation. A score of ≥ 8 indicates the presence of reflux. All patients underwent preoperative gastroscopy, with some patients completing gastroscopic examinations during follow-up.

### Animal model

Thirty 12-week-old male Goto-Kakizaki (GK) rats were obtained from the Shanghai Laboratory Animal Center of the Chinese Academy of Sciences, China, and they were housed in a specific pathogen-free room at the Experimental Animal Center of the Fudan University School of Medicine under a 12-h light/dark artificial circadian rhythm, with sterile water and rat feed provided ad libitum. After one week of acclimation, the rats were randomly assigned into three groups: SG, GGCP + Nissen, and Sham groups. There were 10 rats assigned per group. For the GGCP + Nissen group, the gastric fundus is wrapped around the esophagus from left to right and secured with two 5–0 Prolene sutures to the left gastric wall, with the first suture anchoring the esophageal muscle layer. The stomach was sutured using 5–0 Prolene from the point 3–5 mm distal to the pylorus until the fundus. For the SG group, a toothed vascular clamp was then applied along the greater curvature, and resection of three-quarters of the gastric body was initiated 3–5 mm adjacent to the pylorus, proceeding along the clamp toward the inferior aspect of the His angle to ensure complete fundic excision. The resection margin was subsequently closed in a continuous suture using 5–0 Prolene sutures for seromuscular reinforcement. For the sham group, the stomach, esophagus, and small intestine were surgically exposed, yet no additional procedures were performed. Rats were under isoflurane/O2 mixture anesthesia.

Oral glucose tolerance tests (OGTT) were performed 6 weeks after surgery. GK rats were fasted for 12 h, at which point a 50% glucose solution (3 g/kg body weight) was administered via gastric lavage. Blood glucose levels were measured via tail vein blood collection at 0, 15, 30, 60, 90, 120 and 180 min after gastric lavage. For the clamp test, rats were fasted for 12 h and anesthetized. The carotid arteries and veins are cannulated, and the clamp technique was used to evaluate insulin resistance and pancreatic secretions.

### Statistical analysis

Comparison of mean values was performed using Student’s t-test for normally distributed data. Nonparametric statistics, such as the Mann–Whitney U test, were used to compare ordinal variables and continuous variables with abnormal distributions. In addition, weight and blood glucose levels in animal study were compared between the two surgical groups using a two-way ANOVA and a post hoc Sidak test for multiple comparisons. Statistical significance was set at *P* < 0.05.

## Results

### Patient characteristics and long-term outcomes

During the study period, 35 patients underwent GGCP + Nissen and 40 patients underwent SG. The patient characteristics are listed in Table 1. The mean BMI and weight were 29.42 ± 2.42 kg/m^2^ and 80.38 ± 10.58 kg in the GGCP + Nissen group, respectively, while the corresponding values were 30.35 ± 1.24 kg/m^2^ and 82.58 ± 6.83 kg in the SG group, respectively. At baseline, no patients in the SG group had GERD. In the GGCP + Nissen group, eight patients had symptoms of reflux.

**Table 1:** Patient demographics

	GGCP + Nissen group (*N* = 35)	SG group (*N* = 40)	*P*-value
Age	32.09 ± 8.54	33.28 ± 7.70	*P* > 0.05
Men	*N* = 4	*N* = 4	
Women	*N* = 31	*N* = 36	
Weight, kg	80.38 ± 10.58	82.58 ± 6.83	*P* > 0.05
BMI, kg/m^2^	29.42 ± 2.42	30.35 ± 1.24	*P* > 0.05
Total cholesterol, mmol/L	4.97 ± 0.92	5.01 ± 1.00	*P* > 0.05
HDL cholesterol, mmol/L	1.29 ± 0.30	1.06 ± 0.19	*P* < 0.001
LDL cholesterol, mmol/L	3.11 ± 0.78	3.02 ± 0.11	*P* > 0.05
Triglycerides, mmol/L	1.57 ± 1.09	3.22 ± 4.18	*P* < 0.05
Diastolic blood pressure, mm Hg	80.10 ± 11.48	80.36 ± 9.476	*P* > 0.05
Systolic blood pressure, mm Hg	122.6 ± 14.42	127.6 ± 12.63	*P* > 0.05

**Fig. 2:**
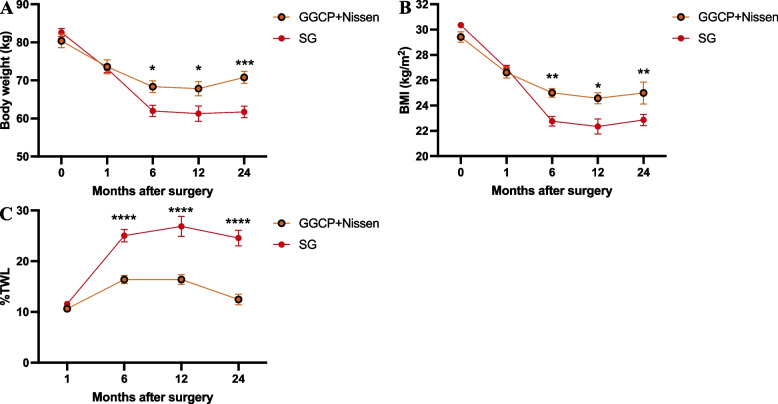
Weight loss parameters. **A** Body weight (kg). **B** BMI (kg/m^2^). **C** % TWL. ****P < 0.0001, ***P < 0.001, ** *P* < 0.01, *P < 0.05 GGCP+Nissen vs SG

The GGCP + Nissen group’s patients followed up at 1, 6, 12, and 24 months postoperatively; there were respectively, 26, 24, 24, and 32 patients following up at those intervals. As for the SG group, at 1, 6, 12, and 24 months, patient follow up was respectively 33, 36, 30, and 33. As shown in Table 2, these two surgical procedures both have significant weight loss effects within one year postoperatively. SG yielded higher 6-month %TWL (25.05 ± 7.24 vs 16.38 ± 3.69, *P* < 0.05), 12 months %TWL (26.85 ± 9.13 vs 16.40 ± 4.96, *P* < 0.05), and 24 months %TWL (24.57 ± 8.61 vs 12.46 ± 5.90, *P* < 0.05) compared to the GGCP + Nissen group. Over the course of two years postoperatively, the disparity between the two groups becomes increasingly pronounced. However, there was no significant difference in %TWL at the 1-month follow-up after surgery between the GGCP + Nissen and SG groups (*P* > 0.05) (Table 2) (Fig. 2). After two years postoperatively, 11 individuals in the GGCP + Nissen group had a %TWL of less than 10%, whereas in the SG group, only one person had a %TWL of less than 10%. This indicates that in terms of long-term weight loss outcomes, GGCP is inferior to SG surgery.

**Table 2:** Weight loss parameters

	Post operation duration	GGCP + Nissen group	SG group	*P*-value
Body weight (kg)	1 month	73.57 ± 9.38	73.09 ± 6.11	*P* > 0.05
	6 months	68.36 ± 7.07	61.97 ± 8.65	*P* < 0.05
	12 months	67.85 ± 9.14	61.30 ± 9.38	*P* < 0.05
	24 months	70.80 ± 9.10	61.70 ± 8.54	*P* < 0.05
BMI, kg/m^2^	1 month	26.6 ± 2.22	26.94 ± 1.42	*P* > 0.05
	6 months	25.00 ± 1.54	22.77 ± 2.22	*P* < 0.05
	12 months	24.57 ± 2.15	22.34 ± 2.69	*P* < 0.05
	24 months	24.99 ± 5.02	22.87 ± 2.52	*P* < 0.05
% TWL	1 month	10.64 ± 2.86	11.55 ± 2.83	*P* > 0.05
	6 months	16.38 ± 3.69	25.05 ± 7.24	*P* < 0.05
	12 months	16.40 ± 4.96	26.85 ± 9.13	*P* < 0.05
	24 months	12.46 ± 5.90	24.57 ± 8.61	*P* < 0.05

In the GGCP + Nissen.group, all 8 of the patients who experienced reflux prior to surgery exhibited postoperative resolution of reflux symptoms. Conversely, regarding the SG group, 10 patients suffered from new onset reflux 1 month after surgery; at the 24 months mark after surgery 4 of the sleeve patients still still were complaining of persistent reflux symptoms (Table 3). This suggests that GGCP + Nissen is effective at mitigating reflux symptoms and possibly preventing gastroesophageal reflux. The SG group demonstrated asymptomtic pre-operative patients suffering from new onsest post-operative reflux. The sleeve group failed to demonstrate any evidence of reflux prevention or reflux mitigation/resolution.

**Table 3:** Reflux before and after surgery

	Pre-operation	1 month	6 months	12 months	24 months
GGCP + Nissen group	8/35	2/26	0/24	0/24	0/32
SG group	0/40	10/33	5/36	4/30	4/33

A GERD-Q score of ≥ 8 indicates the presence of reflux

### Operative outcomes and short-term complications of the clinical cohort

**Fig. 3:**
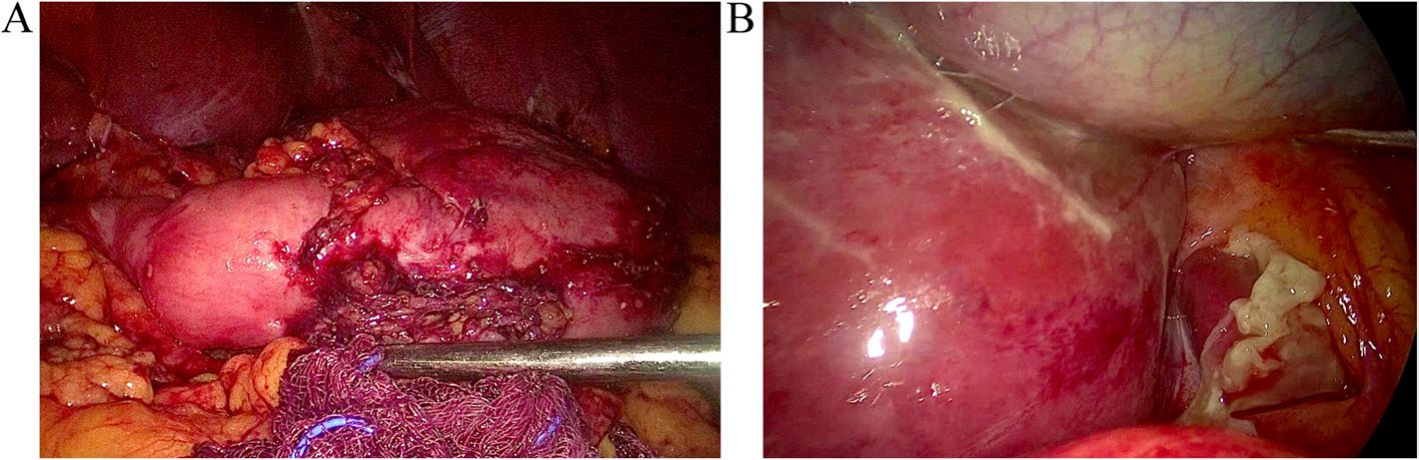
Gastric leakage under laparoscopy. **A** stomach. **B** fundus of stomach

The mean operative times were 132.3 ± 24.83 and 86.9 ± 16.93 min in the GGCP + Nissen and SG groups, respectively. All procedures were performed laparoscopically, and no intra-operative complications were observed. The mean hospitalization stay was 5.5 ± 1.94 days and 5.54 ± 1.39 days in the GGCP + Nissen and SG groups, respectively. The most common short-term complication was nausea and vomiting. During the first 30 days, 16 and 17 patients experienced nausea and vomiting in the GGCP + Nissen and SG groups, respectively. One patient in the GGCP + Nissen group required emergency room visits and reoperation due to a leak (Fig. 3). This complication was treated with laparoscopic drainage and GGCP revision. During the reoperation, upon laparoscpic examination of the stomach, there was evidence of gastric leakage. Nevertheless, the specific site of gastric leakage was not identified.

### Outcomes of the animal model

**Fig. 4:**
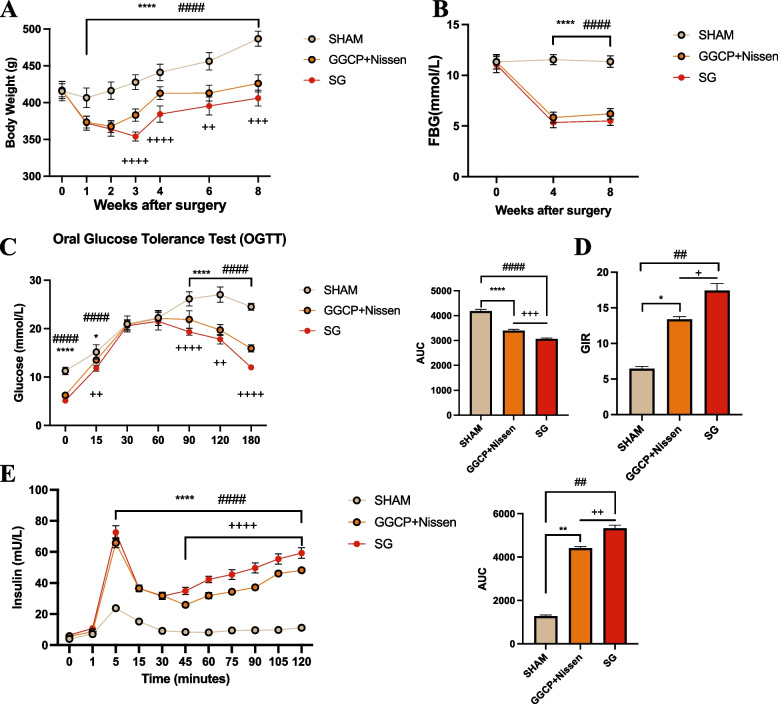
The improvement of glucose homeostasis in animal model. **A** Body weight after surgery. **B** Fasting blood-glucose after surgery. **C** Oral glucose tolerance after surgery. **D** Glucose infusion rate after surgery. **E** insulin level. ****P < 0.0001, ***P < 0.001, ** *P* < 0.01, *P < 0.05 SHAM vs GGCP+Nissen; #### *P* < 0.0001, ### *P* < 0.001, ## *P* < 0.01, # *P* < 0.05 SHAM vs SG. ++++ *P* < 0.0001, +++ *P* < 0.001, ++ *P* < 0.01, + *P* < 0.05 GGCP+Nissen vs SG

Thirty GK rats were used in the present study (*n* = 10 per group). No rats died in any group. There was no significant difference in the preoperative weight between the groups; however, post-surgery, both the GGCP + Nissen and SG groups showed significant weight loss compared to SHAM group. However, at 3, 4, 6, and 8 weeks after surgery, the weight of GGCP + Nissen rats was significantly greater than that of SG rats (*P* < 0.05) (Fig. 4A). To study the effects of both GGCP + Nissen and SG on glucose homeostasis, fasting blood glucose levels were monitored after surgery, and the OGTT, clamp test, and hyperglycemic clamp tests were performed to evaluate glucose homeostasis after surgery. Fasting blood glucose significantly improved in both the GGCP + Nissen and SG group compared to SHAM group, with no significant difference between the two treated groups (Fig. 4B). The OGTT was performed 6 weeks after surgery (Fig. 4C). The results showed that blood glucose levels in the sham group were significantly higher than those in the GGCP + Nissen and SG group at 0, 15, 90, 120, and 180 min (*P* < 0.05). In addition, blood glucose levels in the GGCP + Nissen group at 15, 90, 120, and 180 min were significantly higher than those in the SG group (*P* < 0.05). The AUC was calculated for each group, and analysis showed that the AUC of SHAM group was significantly higher than those of the GGCP + Nissen and SG groups (*P* < 0.05), whereas the AUC of the SG group was significantly lower than that of the GGCP + Nissen group (*P* < 0.05). In the clamp and hyperglycemic clamp tests (Fig. 4D-E), glucose infusion rate (GIR) and insulin levels in the SHAM group were significantly lower than those in the GGCP + Nissen and SG group (*P* < 0.05). Furthermore, significant differences in GIR and insulin levels were identified between the GGCP + Nissen and SG group. Overall, these data showed that SHAM group had lower insulin sensitivity and insulin secretion than the treatment groups, while the GGCP + Nissen group showed lower insulin sensitivity and insulin secretion than the SG group. These results indicate that SG led to a significant improvement in blood glucose levels compared to GGCP + Nissen.

## Discussion

Bariatric surgery is a continuously evolving field of medicine. Just over the past decade, some surgical techniques have been abandonedand other procedures have been gaining popularity. SG is widely accepted as an acceptable, standalone bariatric procedure; however, concerns remain regarding the long staple line and the risk of gastric bleeding and leaks [13]. GGCP + Nissen held promise as a reasonable treatment option for patients with concurrent GERD and obesity. Similar to RYGB, the Nissen fundoplication can reduce the reflux symptoms resulting from acid (or alkaline/bilious) exposure in the esophagus [11]. GGCP + Nissen promotes weight loss through a restrictive mechanism, reducing gastric volume by the creation of rows of sutures*.*

In our study, we focused on obese individuals with a BMI around 30 kg/m^2^. Prior research has indicated that the metabolic efficacy of GGCP in managing obesity is comparatively inferior to that of SG [14, 15]. To delve more precisely into the therapeutic utility of GGCP + Nissen within the context of obesity management, we deliberately selected individuals with a BMI around 30 kg/m^2^.This particular group of patients would benefit from a surgical intervention for weight loss leaning to the more conservative and less invasive. GGCP was seemingly a potentially preferable choice in such circumstances. This study sought to investigate this specific cohort and assess the weight loss outcomes. In our study, the mean %TWL in the GGCP + Nissen group was 16.40% at 12 months postoperatively. Thus, this treatment strategy achieved a comparable weight loss to that induced by current drug treatments [16–18]. Although no significant difference in weight loss was observed between SG and GGCP + Nissen groups at the 1-month postoperative interval, the SG cohort demonstrated progressively superior weight reduction at 6-, 12-, and 24-month post-surgery. From these results, we concluded that while both of these procedures resulted in significant weight loss over the follow-up period, the SG procedure showed a higher weight loss rate, especially at 6-, 12-, and 24-month postoperatively. The comparable 1-month weight loss outcomes likely reflect protocol-driven caloric restriction during the initial recovery phase. This progressive divergence suggests SG's enhanced metabolic efficacy, potentially mediated by sustained hormonal adaptations. Conversely, in this study, the addition of Nissen fundoplication to the GGCP was the only factor shown to mitigate reflux symptoms. Overall, the eight patients in the GGCP + Nissen group with GERD before surgery achieved complete resolution of reflux symptoms after surgery. There was no documentation of any GGCP + Nissen patients exhibiting symptoms of reflux postoperatively during follow-up. Conversely, none of the patients in the SG group had reflux symptoms before the operation. However, 10 patients developed reflux symptoms 1 month after the operation; meanwhile, 4 patients still presented with reflux symptoms 24 months after their SG. We therefore concluded that GGCP + Nissen is an effective procedure for managing obese patients with a BMI around 30 kg/m^2^ and concomitant GERD—yet this weight loss is not as profound as with SG. As an effective anti-reflux procedure, Nissen fundoplication can be combined not only with GGCP but also with SG to reduce postoperative reflux following SG [19]. Future studies should further explore and optimize the synergistic application of multiple surgical techniques to achieve enhanced clinical outcomes.

It is of note that one patient in the GGCP + Nissen group experienced a serious complications requiring reoperation. On the other hand, there were no serious complications noted in the SG group. This situation may be related to potential damage to the gastric wall structure during surgical procedures and increased intra-gastric pressure postoperatively. Perforation incidents have also been observed postoperatively in surgeries combining SG with Rossetti fundoplication. The reasons behind both occurrences may be similar [20]. Nausea and vomiting were the most common early postoperative complications in patients in both groups. Postoperative nausea/vomiting after surgery often stems from anesthesia stimulating the vomiting center, disrupted gut motility, or surgical nerve irritation. These symptoms were medically controlled and most patients recovered within a few days.

Considering the uncertainty regarding the efficacy of GGCP + Nissen at achieving glycemic improvement, we did not include patients with severe metabolic complications or diabetes in the group. So we did not investigate this factor in patients with obesity and diabetes. Instead, to compare the effects of GGCP + Nissen and SG on glucose homeostasis, a rat model of diabetes was established. Compared with SHAM group, the GGCP + Nissen group showed significant improvements in glucose tolerance, fasting blood glucose, insulin resistance, and increased insulin secretion. These results suggest that GGCP + Nissen improves glucose homeostasis along with obesity. However, in this study, GGCP + Nissen was noted to be inferior to SG in terms of improving fasting blood glucose, glucose tolerance, insulin resistance, and increased secretion of insulin. This effect may stem from SG-induced enhancements in gastric emptying and the regulation of key gastrointestinal hormones, including ghrelin, polypeptide YY, and glucagon-likepeptide-1 [21]. Ghrelin, a peptide hormone predominantly secreted by the gastric fundus, plays a critical role in appetite regulation and metabolic homeostasis. SG, which involves resection of the gastric body and fundus, has been shown to significantly reduce ghrelin production. In contrast, GGCP + Nissen preserves the fundus. Notably, endoscopic gastric plication – a procedure mechanistically analogous to GGCP + Nissen – has demonstrated paradoxical increases in ghrelin secretion according to recent studie [22]. This fundamental distinction in gastrointestinal hormonal modulation may underlie the superior weight loss and glycemic control outcomes observed with SG compared to GGCP + Nissen procedures.

This study has several limitations that should be acknowledged. First, its retrospective design and relatively short follow-up duration may introduce selection bias and limit the assessment of long-term outcomes. Second, the modest sample sizes reduce statistical power to detect subtle intergroup differences and constrain generalizability to broader populations. Third, the analysis did not incorporate psychological or quality-of-life assessments or systematically evaluate nutritional complications such as micronutrient deficiencies. To address these gaps, future investigations should prioritize prospective randomized controlled trials with extended follow-up periods to comprehensively compare the durability, metabolic effects, and safety profiles of GGCP + Nissen versus established bariatric procedures like sleeve gastrectomy and Roux-en-Y gastric bypass.

## Conclusions

This retrospective clinical and animal study demonstrated that GGCP + Nissen effectively aided in obesity management and glucose metabolism. However, compared with SG, GGCP + Nissen was inferior at treating obesity and inferior in glucose homeostasis. In terms of symptomatic reflux management, the GGCP + Nissen demonstrated the ability to treat clinically significant reflux. Conversely, in the SG cohort, a consequential proportion of patients, despite their significantly better weight reduction parameters, seemed to suffer from post-surgical reflux.

## Data Availability

The datasets used and/or analysed during the current study are available from the corresponding author on reasonable request.

## Abbreviations

SG: Sleeve gastrectomy
RYGB: Roux-en-Y gastric bypass
GGCP: Gastric greater curvature plication
GERD: Gastroesophageal reflux disease
BMI: Body mass index
%TWL: Percentages of total body weight loss
GERD-Q: GERD-questionnaire
GK: Goto-Kakizaki
OGTT: Oral glucose tolerance tests
GIR: Glucose infusion rate
